# The Effects of Remote Consultation (RC) on Outpatient Clinic Attendance Rates in City Community Mental Health Team (CMHT) and Patient Feedback on RC

**DOI:** 10.1192/bjo.2022.403

**Published:** 2022-06-20

**Authors:** Ting Miller, Joshua Thomas, Anitha Mukundan

**Affiliations:** 1 Leeds and York Partnership NHS Foundation Trust, Leeds, United Kingdom; 2 Bradford District Care Trust, Bradford, United Kingdom

## Abstract

**Aims:**

The outbreak of COVID-19 in 2020 forced a sudden change in service delivery in CMHT. Remote consultations (RC) via telephone or video were introduced to facilitate safe contact between staff and patients. Traditional face to face (F2F) appointments have high rates of non-attendance (DNA). This project aimed to examine whether the DNA rate for CMHT appointments has been affected by the introduction of RC. In addition to this, patients were asked to give feedback about how they felt about the use of RC.

**Methods:**

We retrospectively studied the outcome of outpatient medical appointments within City CMHT over two periods, namely pre COVID-19 which was between April to June 2019 and during COVID-19 which corresponded to the same period in 2020. A list of patients over these two periods were extracted from trust electrical medical record: System One (S1). Further review patients’ notes on S1 was conducted to identify DNA group, among which detailed information including gender, age groups, types of outpatient clinics (urgent or routine, first review or follow-up review), types of consultations (remote or F2F).

In addition, an anonymous patient feedback form on RC was given out to 30 patients attending F2F appointments at the clinic between May and August 2021.

**Results:**

94% appointments were conducted remotely in 2020 while 100% were F2F in 2019 during the periods studied. 2020 saw a 16% increase in attendance rate and a nearly half reduction in cancelled appointments from 30% to 16%. There was a slight drop in DNA rate by 2%.

19 patient feedbacks indicated at least one RC experience. Among them, 47% rated it as very good and 58% felt RC offered the same level of care and treatment as F2F. On the other hand, 74% would like to be seen F2F for future appointments when given a choice.

Free comments about RC were captured including ‘Not everything gets covered’, ‘it makes me anxious to talk to a medical team over the phone’ and ‘things like bruises could be missed in a RC’. However, one patient said they found RC is less stressful.

**Conclusion:**

A massive shift from F2F to RC was seen due to COVID-19 restriction. Attendance rated was improved with RC, however, it was mainly achieved by a significant reduction in cancelled appointments. Its impact on overall DNA rate appeared minuscule.

Despite nearly half of the patients indicated RC is as good as F2F. Most patients prefer f2f for future consultation.

